# Naturalistic psychedelic therapy: The role of relaxation and subjective drug effects in antidepressant response

**DOI:** 10.1177/02698811241278873

**Published:** 2024-09-20

**Authors:** Abigail E Calder, Benjamin Rausch, Matthias E Liechti, Friederike Holze, Gregor Hasler

**Affiliations:** 1 Molecular Psychiatry Lab, Faculty of Science and Medicine, University of Freiburg, Villars-sur-Glâne, Switzerland; 2 Faculty of Pharmacy, Saarland University, Saarbuecken, Germany; 3 Clinical Pharmacology, Department of Clinical Research, University Hospital Basel and University of Basel, Basel, Switzerland; 4 Lake Lucerne Institute, Vitznau, Switzerland; 5 Freiburg Mental Health Network, Chemin du Cardinal-Journet 3, Villars-sur-Glâne, Switzerland

**Keywords:** Psychedelic-assisted therapy, LSD, psilocybin, psychedelics, mystical experience, relaxation, depression, adverse effects

## Abstract

**Background:**

Psychedelic-assisted therapy (PAT) is permitted in Switzerland under its limited medical use program. Data from patients in this program represent a unique opportunity to analyze the real-world practice of PAT.

**Aims:**

This study compared the subjective effects of lysergic acid diethylamide (LSD) and psilocybin between patients undergoing PAT and healthy volunteers. For the patients, it also investigated the relationship between antidepressant effects and six measures of acute drug effects.

**Methods:**

We compared data on acute psychedelic drug effects between 28 PAT patients with data from 28 healthy participants who participated in a randomized, double-blind crossover trial. All participants received varying doses of psilocybin and LSD. Subjective effects were assessed on an hourly basis during the acute drug effects, and the Mystical Experience Questionnaire (MEQ) was completed retrospectively. For patients, depressive symptoms were assessed using the Montgomery-Åsberg Depression Rating Scale (MADRS).

**Results:**

Ratings of overall drug effect and mystical experience were similar across groups. Compared with healthy controls, patients reported lower ratings of ego dissolution. Patients showed a significant decrease in MADRS scores, and the greatest predictor of antidepressant outcome was relaxation during the PAT session. We did not observe a relationship between mystical-type experiences and antidepressant effects. Most patients experienced mild adverse effects which resolved within 48 h.

**Conclusion:**

PAT reduced depressive symptoms in this heterogeneous patient group. Patients may experience more challenging psychedelic effects and reduced ego dissolution. Hourly assessment of drug effects may predict clinical outcomes better than retrospectively assessed mystical experiences, and the impact of relaxation during PAT should be investigated further.

## Introduction

Psychedelic-assisted therapy (PAT) with psilocybin and lysergic acid diethylamide (LSD) has demonstrated efficacy in the treatment of depression, anxiety, and some other neuropsychiatric disorders (Gasser et al., 2014; Goodwin et al., 2022; Holze et al., 2023b; Raison et al., 2023; von Rotz et al., 2023). Given promising preliminary data, PAT has been conducted in Switzerland under its limited medical use program from 1988 to 1993, and again since 2014 (Calder and Hasler, 2023a). This program allows PAT with LSD, psilocybin, or the empathogen 3,4-methylenedioxymethamphetamine (MDMA) under the supervision of a licensed physician for patients with an applicable treatment-resistant diagnosis. Eligible diagnoses include post-traumatic stress disorder (Mitchell et al., 2023), major depressive disorder (Galvao-Coelho et al., 2021), anxiety disorders (Holze et al., 2023b), substance use disorders (Bogenschutz et al., 2022), obsessive-compulsive disorder (Moreno et al., 2006), eating disorders (Calder et al., 2023), and others. Patients may undergo multiple sessions if needed, typically at intervals of several weeks or months, and this flexible dosing schedule is an important difference between naturalistic PAT and controlled trials. Patients are also not required to taper off psychiatric medications before undergoing PAT when the risk of unfavorable drug interactions appears low (Becker et al., 2022; Malcolm and Thomas, 2022). In addition, patients who positively responded to PAT in clinical trials sponsored by academia or industry may continue PAT within the limited medical use program, fulfilling recent recommendations for ethical continuing care after clinical trials of PAT (Jacobs et al., 2024). Data from Swiss PAT reflect real-world variation in substances, doses, diagnoses, treatment duration, and concomitant medications, and can thus offer valuable insights into how PAT is already practiced outside of controlled clinical trials.

Many PAT practitioners regard the subjective acute psychedelic effects as important for the therapeutic outcome (Yaden and Griffiths, 2021). However, there is some debate about precisely which effects are important. One view is that mystical-type experiences, commonly measured at the end of a PAT session with the Mystical Experience Questionnaire (MEQ), are particularly important for therapeutic effects (Griffiths et al., 2006, 2016; Liechti et al., 2017; Ross et al., 2016). A “complete” mystical experience involves feelings of profound unity and sacredness, euphoria, and transcendence of time and space, as well as a sense of experiencing profound truth one struggles to capture in words (i.e., ineffability; Barrett et al., 2015). The association between mystical-type experiences and clinical outcomes has been shown in studies of psilocybin-assisted therapy for depression, end-of-life distress, and substance use disorders, as well as in LSD-assisted therapy for anxiety (Holze et al., 2023b; Ko et al., 2022). However, other studies have not found associations between symptom improvement and mystical-type experiences (Agin-Liebes et al., 2020; Gukasyan et al., 2022; Sloshower et al., 2023). Some scholars argue that the acceptance of mystical-type experiences as a defining therapeutic mechanism in PAT is too hasty and that the role of other psychedelic effects is in danger of being overlooked (Sanders and Zijlmans, 2021).

Previous research has also investigated whether the intensity of pleasant and unpleasant psychedelic effects is related to therapeutic outcomes. One meta-analysis found that the overall intensity of a psychedelic experience was the best predictor of therapeutic effects (Romeo et al., 2021). In general, overall positively experienced psychedelic effects also seem to correlate positively with long-term therapeutic outcomes (Holze et al., 2023b; Roseman et al., 2017). The pattern is less clear for acutely unpleasant effects or “challenging experiences,” whose relationship with therapeutic efficacy likely depends on how they are resolved (Carhart-Harris et al., 2018). In addition, the experience of ego dissolution is considered important in PAT by some practitioners. Ego dissolution is the temporary full or partial cessation of the subjective sense of self (Nour et al., 2016). Many consider it to be a typical effect of both LSD and psilocybin (Mason et al., 2020; Tagliazucchi et al., 2016), particularly at higher doses (Holze et al., 2021). In supportive settings, ego dissolution appears to be related to subsequent reductions in symptoms of depression and anxiety (Kiraga et al., 2022; van Oorsouw et al., 2022).

Mystical-type experiences, ego dissolution, and other psychedelic drug effects are usually measured retrospectively, after a return to normal consciousness (Schmid et al., 2021; Schmidt and Berkemeyer, 2018). However, it is also feasible to obtain measures of psychedelic drug effects in real time using relatively simple measures in the form of visual analog scales (Holze et al., 2022b) or verbal Likert scales (Vogt et al., 2023). Many studies in healthy volunteers have asked participants to rate the intensity of psychedelic effects at regular intervals during the experience, including the overall intensity of drug effects, positive and negative effects, and ego dissolution (Holze et al., 2021; Ley et al., 2023; Straumann et al., 2023; Vogt et al., 2023). Real-time data may be valuable in clinical settings as well, for two main reasons. First, there can be significant variation in how people view an experience while it is happening and after it has ended (Oishi, 2016). It is thus interesting to assess whether real-time ratings correlate with therapeutic outcomes in the same way that retrospective ratings, such as MEQ scores, appear to do. Notably, previous research suggests that retrospectively measured effects of LSD at 10 or 24 h post-dosing were very similar to those measured during the peak response at 3 h (Liechti et al., 2017). Second, real-time ratings can give PAT practitioners valuable and immediate feedback on how a psychedelic experience is progressing in a clear and minimally intrusive way while there is still time to influence the experience if necessary. People undergoing psychedelic treatments may not spontaneously articulate how they are feeling, and simple verbal feedback about the overall intensity, valence, and other effects can provide valuable information about the progress and nature of patients’ experiences and the therapeutic process.

In the present study, we analyze naturalistic data from PAT with psilocybin and LSD from the Swiss limited medical use program together with data from a randomized controlled crossover trial with psilocybin and LSD in healthy participants (Holze et al., 2022b). In the patient group, we investigate dose–response relationships of different doses of LSD and psilocybin on subjective effects, as well as the relationship between subjective drug effects and depressive symptoms. In addition, we investigate whether subjective effects differ between patients and healthy participants receiving the same well-characterized drug formulation and dose (Holze et al., 2019, 2023a), as well as the relationship between real-time assessments of subjective effects and retrospective MEQ scores. Finally, we report the adverse effects of PAT in the patient group. These data offer valuable insights into PAT in real-world clinical practice outside of controlled trials.

## Methods

### Design

**Figure 1.**
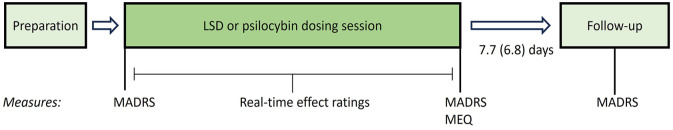
Procedures and assessments in the patient group undergoing psychedelic-assisted therapy (PAT). PAT consisted of two preparatory consultations, one dosing session, and one follow-up consultation. Individual patients could repeat PAT at most once every 3 months. MADRS: Montgomery-Åsberg depression rating scale; MEQ: mystical experience questionnaire.

Patients were treated with PAT at the Freiburg Network for Mental Health in Freiburg, Switzerland, after either self-referral or referral by another physician or therapist. PAT was approved by the Swiss Federal Office for Public Health. Each round of PAT consisted of two preparatory consultations, one dosing session, and 1–3 post-dose integration sessions (mean: 1.6) as needed (Figure 1). Briefly, the first preparatory session included a full psychiatric history, a discussion of current complaints, and a discussion about the indication and appropriateness of PAT for each patient. In addition, patients were informed about the range of positive and negative effects they might experience in PAT and invited to discuss their goals for the therapy session. The second preparatory session included a discussion of practical concerns, as well as discussions of what to expect from the therapeutic environment and how any challenging effects of LSD or psilocybin may be managed. Integration sessions were used to discuss therapeutically relevant content that arose during PAT, as well as strategies for facilitating lasting therapeutic improvement. The first integration session always took place within 48 h of dosing. Patients were allowed to repeat PAT sessions as needed, but at most once every 3 months. All patients were additionally attending long-term psychotherapy (CBT, ACT) with either the treating psychiatrist (GH) or an external psychiatrist or psychotherapist.

Healthy participants were recruited for a double-blind, placebo-controlled crossover trial investigating the dose equivalence of LSD and psilocybin. Study procedures are described in detail elsewhere (Holze et al., 2022b). Briefly, each participant attended one screening visit and five dosing days with two doses each of LSD and psilocybin on separate days, as well as a placebo day which was not used for the current analysis. Doses were given in random order and at least 10 days passed between each dosing day. During the acute psychedelic effects, participants were attended by at least one investigator. Data on subjective effects and side effects were collected within the first 24 h after dosing, after which participants were discharged home. The study was approved by the Ethics Committee of Northwest Switzerland and the Swiss Federal Office for Public Health.

### Participants

Twenty-eight patients underwent a total of 55 open-label treatment sessions with psilocybin or LSD between January 2020 and May 2023 (Table 1). All patients provided written informed consent for their data to be used. Psychiatric diagnoses were determined through clinical interviews, utilizing the Diagnostic and Statistical Manual of Mental Disorders, Fifth Edition (DSM-5) criteria. The sample was composed entirely of residents of Switzerland (50% female), and the average age upon entering treatment was 49 years (Table 1). Exclusion criteria for PAT included personal and/or family history of psychotic disorders, bipolar I disorder, borderline personality, epilepsy, dementia, and pregnancy. Only two patients had previous experience with psychedelics before entering PAT, both with psilocybin mushrooms.

**Table 1.** Patient characteristics for *N* = 28 patients undergoing psychedelic-assisted therapy. Age and number of treatment sessions are expressed as mean (SD). Further patient data are in Table S1.

ID	Sex	Age	Diagnosis	No. PAT sessions
LSD1	F	34	MDD, BN	2
LSD2	F	64	MDD	1
LSD3	F	43	MDD	3
LSD4	F	40	Bipolar II	2
LSD6	M	51	MDD, PTSD	1
LSD7	M	54	MDD	1
LSD8	F	61	MDD	1
LSD9	F	58	MDD	2
LSD10	F	44	MDD	5
LSD11	F	61	MDD	1
LSD12	F	48	MDD, AN	1
LSD13	F	59	MDD, ADD	3
LSD14	M	23	OCD	4
LSD15	F	59	Social anxiety	1
LSD16	F	34	MDD, AN	1
LSD17	M	54	MDD, AUD, ADD	1
PSI1	F	61	MDD, GAD, ASD	1
PSI2	M	21	MDD	2
PSI3	M	35	OCD	2
PSI4	M	49	MDD	5
PSI5	M	72	MDD	1
PSI6	F	51	MDD	1
PSI7	M	35	MDD	2
PSI8	M	63	MDD	3
PSI9	M	53	MDD, GAD	2
PSI10	M	19	MDD	1
PSI11	M	78	MDD	3
PSI12	M	42	MDD	2
Summary	14F, 14M	48.79 (14.87)		1.96 (1.18)

ADD: attention deficit disorder; AN: anorexia nervosa; ASD: autism spectrum disorder; AUD: alcohol use disorder; BN: bulimia nervosa; GAD: generalized anxiety disorder; MDD: major depressive disorder; OCD: obsessive-compulsive disorder; PTSD: post-traumatic stress disorder.

All patients were diagnosed with a treatment-resistant condition for which preliminary safety and efficacy of PAT have been demonstrated. Treatment resistance was defined as having attempted at least three different standard treatments, including antidepressants, psychotherapy, or inpatient treatments, with insufficient or absent response. Twenty-four patients (86%) were diagnosed with treatment-resistant major depressive disorder, and the remaining patients were diagnosed with obsessive-compulsive disorder (*N* = 2), bipolar disorder (*N* = 1), or social anxiety (*N* = 1) which had not responded to other treatments.

A relatively high number of patients (78.6%) were taking psychiatric medications either daily or as needed. 60.7% of patients were on at least one antidepressant (SSRIs = 35.7%, bupropion = 21.4%, SNRIs = 7.1%, tricyclic antidepressants = 7.1%). Antipsychotics for the treatment of depression were present in 25% of patients, and 28.6% were taking benzodiazepines. Other medications included anticonvulsants (10.7%), opioids, lithium, and zolpidem (each 7.1%), and thyroid hormone, lisdexamfetamine, and pramipexole (each 3.6%). Most patients did not taper off their medication entirely but skipped any scheduled doses on the morning of PAT to avoid acute medication interactions. In addition, five patients (17.9%) were taking medications to control high blood pressure, which they continued to take during PAT.

Healthy participants were recruited from a participant pool or via word of mouth, and details of the participation criteria can be found elsewhere (Holze et al., 2022b). Briefly, the sample included 28 participants (50% women) with an average age of 35 years and no history of major psychiatric disorders. Exclusion criteria included current intake of psychiatric medications, history of major psychiatric disorders, physical illnesses, >10 lifetime uses of illicit drugs (except cannabis), and illicit drug use within the 2 months prior to the study or during the study period. Fourteen (50%) had previous experience with psychedelics. All participants provided written informed consent.

### Drugs

In both samples, LSD base (>99% purity, Lipomed AG, Arlesheim, Switzerland) was administered orally in a solution containing 100 µg LSD in 1 mL of 96% ethanol. Psilocybin (99.7% purity, ReseaChem GmbH, Burgdorf, Switzerland) was administered in capsules, with one capsule containing 5 mg psilocybin dihydrate. Both substance formulations were produced according to Good Manufacturing Practice.

### Substance administration

**Figure 2.**
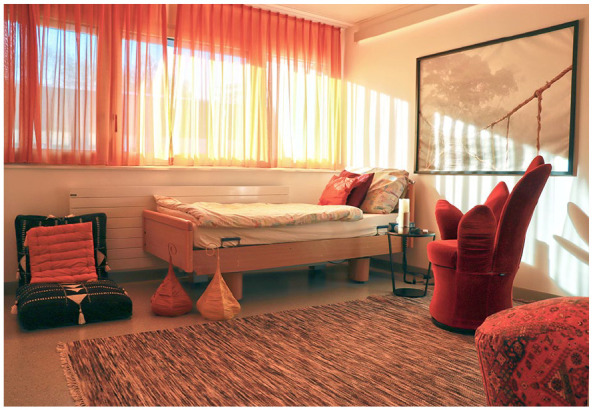
Therapy room at the Fribourg Network for Mental Health in which PAT sessions are conducted.

Patients underwent PAT in a quiet and aesthetic treatment room (Figure 2). LSD or psilocybin was administered in the morning between 8:00 and 10:00. The standard dose to start the treatment was 100 µg LSD or 15 mg psilocybin, and doses could be adjusted based on experiences in prior sessions. In 16/55 PAT sessions, the first dose did not achieve the desired effect and a second dose of the same drug was administered after 1–3 h. The desired effect was defined as a score of at least 40/100 on the rating of drug intensity, though clinical judgment was also used to decide whether a patient would benefit from a second dose or not. The second dose was either 50 or 100 µg LSD or 5 or 10 mg psilocybin, depending on response to the first dose. This flexibility was necessary because the effects of psychiatric medications on psychedelic drug effects are still only partially understood, in addition to the fact that individuals vary in their sensitivity to psychedelics (Halman et al., 2023). After 2–3 months, patients were allowed to discuss the option of further PAT sessions with the treating psychiatrist. Decisions about whether to continue, as well as with which substance and dose, were based on the therapeutic effects and side effects from the previous session, as well as patient preferences and responses to alternative treatments. Total doses for individual PAT sessions ranged from 100 to 250 µg LSD (median: 100 µg) and 10–25 mg psilocybin (median: 20 mg).

During PAT sessions, patients were encouraged to relax and wear eyeshades while listening to a pre-programmed music playlist, which also included periods of silence. When agreeable to the patient, the psychiatrist led a guided meditation immediately following substance administration; patients were also allowed to read or sit quietly and listen to music while waiting for the onset of effects. The psychiatrist (GH) was present in the treatment room during all sessions and obtained real-time ratings of acute drug effects once per hour. Patients were allowed to return home that evening accompanied by a friend or family member once psychedelic effects had sufficiently dissipated (approximately 6 h after psilocybin treatment and 9 h after LSD treatment).

Healthy participants received LSD or psilocybin at 9:00 in the morning in a calm hospital room. One investigator remained present during the sessions, and participants were permitted to listen to music, converse with the investigator, or do other quiet activities compatible with study procedures. Various outcome measures, including real-time ratings of acute effects, were assessed at least once per hour for up to 16 h. Participants were allowed to return home 24 h after drug administration.

### Measures

Real-time ratings of acute drug effects were obtained hourly in patients, and at least hourly in healthy participants. Patients were verbally asked to rate the intensity of five acute effects on a scale from 0 to 10, including the overall effect intensity (“How strong are the effects?”), good drug effects (“How strong are good drug effects?”), bad drug effects (“How strong are bad drug effects?”), ego dissolution (“How strong is the blurring of the boundary between me and my surroundings?”), and relaxation (“How relaxed do you feel?”). The rating scale was explained before dosing to make answering the questions as easy as possible. Healthy subjects were asked to rate the same effects, except relaxation, using pencil-and-paper visual analog scales and a scale from 0 to 100. Patient scores were converted to a 0–100 scale for comparability between samples. After the dosing sessions, participants also completed the German version of the MEQ30 (Liechti et al., 2017). MEQ scores were available for all healthy controls and for 41 out of the 55 PAT sessions. “Complete” mystical experiences were defined as a score of >60% of the total possible score on all four subscales of the MEQ30 (Barrett et al., 2015).

To assess rapid antidepressant effects, the Montgomery-Åsberg Depression Rating Scale (MADRS) was completed at baseline and immediately after each PAT session before patients returned home. The treating psychiatrist rated depression symptom severity using the MADRS before and after the dosing day. In addition, in 31 out of 55 sessions, patients provided follow-up MADRS scores on average 7.7 days (SD: 6.8) after the session, referring to their symptoms in the days since the session. The standard look-back period of 1 week was used for all MADRS timepoints except for the MADRS at the end of the dosing day, which was used only to assess depressive symptoms on that day. This allowed us to assess rapid antidepressant effects, which typically appear immediately after PAT (Kadriu et al., 2021).

Adverse effects were systematically assessed after PAT sessions using a preliminary version of the Swiss Psychedelic Side Effects Inventory (SPSI), which was developed in our clinic by GH and AEC and will be described in detail in an upcoming publication (Calder and Hasler, 2024). Adverse effect assessments at our clinic began in October 2021, and data are available for 31 out of 55 PAT sessions. We drew on questions from several sources, beginning with 42 questions from the List of Complaints which had previously been used with various psychedelics (Holze et al., 2022a; Liechti et al., 2001). We then added 26 additional items to assess more psychedelic-specific side effects, including rare ones. The SPSI additionally asks patients to report how long each symptom lasted, whether it seemed related to PAT, whether it was present before PAT, and whether any medication was needed to alleviate it. In addition, patients rated whether the experience of that symptom was positive or negative and whether that symptom had a positive or negative impact on their life. The treating psychiatrist evaluated side effects using the SPSI at the first integration session and followed up on any persisting adverse effects until their resolution.

### Statistical analyses

Statistical analyses were conducted with RStudio Version 2023.06.1. For real-time ratings of drug effects, we calculated the area under the curve (AUC) of the first 8 h after dosing beginning at zero and using the trapezoidal method. All analyses of acute drug effects and MADRS scores were done using linear mixed-effects models from the lme4 package (V1.1.35.1) (Bates et al., 2015; Dixon, 2003). Subject ID was always included as a random effect to control for the fact that some patients and all healthy subjects were treated multiple times. In analyses comparing LSD and psilocybin, we controlled for the effect of dose by converting psilocybin doses to the LSD-equivalent dose (1 mg psilocybin = 5 µg LSD; Holze et al., 2022b). Welch’s two-sample *t*-test was used to compare the ages between healthy subjects and patients. To reduce the false discovery rate, the Benjamini–Hochberg procedure was applied across all tests.

In the patient sample, some patients received either a second dose of LSD or psilocybin (*N* = 16 sessions) or a sedative (*N* = 3 sessions) between 1 and 3 h after the initial dose. To prevent this from biasing our analyses of acute drug effects, we did not exclude these patients but treated all timepoints after the second dose as missing data. Missing data required for calculating the AUC was imputed using mean imputation by group, drug, and dose. Based on the assumptions of each analysis, we either used the AUCs calculated based on the total dose or on the initial dose (see Supplemental Methods for details).

## Results

### Real-time ratings of subjective effects in patients and healthy subjects

**Figure 3.**
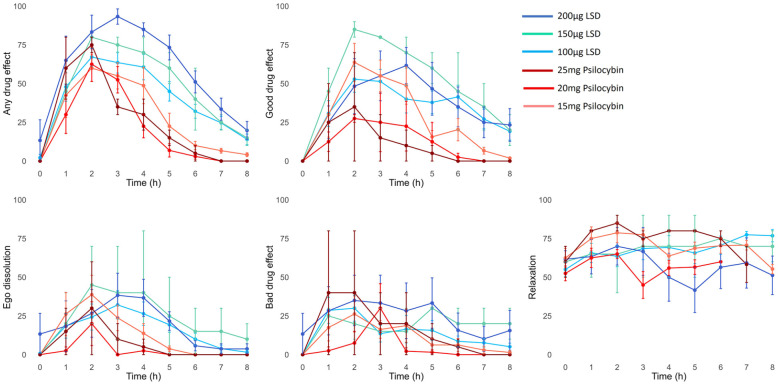
Subjective effects of different doses of LSD and psilocybin in patients. Data are shown as mean ± SEM. *N* = 54 PAT sessions in 28 patients (100 µg LSD = 19, 150 µg LSD = 2, 200 µg LSD = 8, 15 mg psilocybin = 15, 20 mg psilocybin = 7, 25 mg psilocybin = 3). There was a significant dose–response effect for any drug effect (*p* < 0.001). Other dose–response curves are shown for illustrative purposes.

First, we assessed the dose–response relationship for real-time ratings of “any drug effect” in patients who received 100–200 µg LSD or 15–25 mg psilocybin. As expected, real-time subjective effect ratings of “any drug effect” significantly increased with increasing dose (*F*(5, 30) = 6.884, *p* < 0.001) (Figure 3).

**Figure 4.**
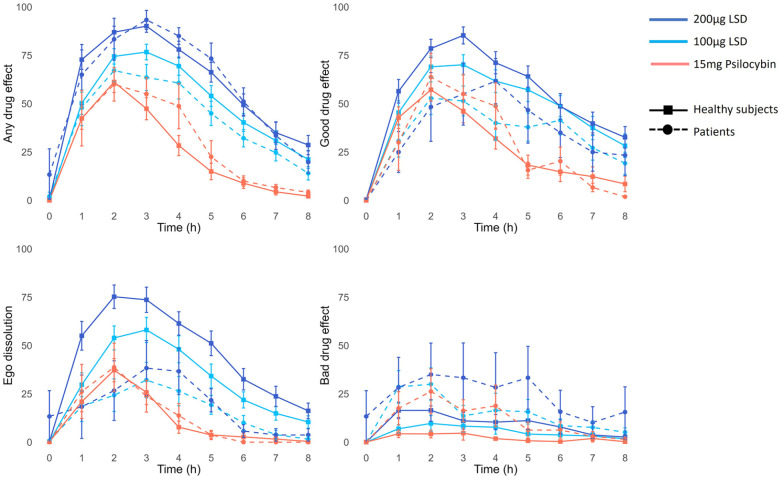
Average real-time ratings of any drug effect, good drug effect, bad drug effect, and ego dissolution at different doses for healthy subjects and patients. Data are shown as mean ± SEM. *N* = 28 healthy subjects for all doses and 42 PAT sessions in 27 patients (100 µg LSD = 19, 200 µg LSD = 8, 15 mg psilocybin = 15). Overall, patients had lower ratings of ego dissolution (*p* < 0.001). The higher ratings of bad drug effects were not statistically significant (*p* = 0.09). There was also no significant difference in any drug effect or good drug effect.

To compare subjective effects between healthy subjects and patients, we used data from all dosing sessions involving 100 µg LSD, 200 µg LSD, or 15 mg psilocybin. Because patients were significantly older than the healthy participants (*t*(45.71) = 4.2, *p* < 0.001), we also included age as a covariate in our model. Patients had significantly lower ratings for ego dissolution (β = −183.50, SE = 36.01, *p* < 0.001) than healthy volunteers, as well as higher ratings of bad drug effect which were not statistically significant (β = 53.70, SE = 21.80, *p* = 0.09) (Figure 4). There were no significant differences between groups in overall drug effect (β = −57.23, SE = 34.08, *p* = 0.30) or good drug effect (β = −98.61, SE = 46.59, *p* = 0.16). In addition, age was significantly and positively related to ego dissolution (β = 4.31, SE = 1.37, *p* = 0.02).

### Mystical experiences in patients and healthy subjects

**Figure 5.**
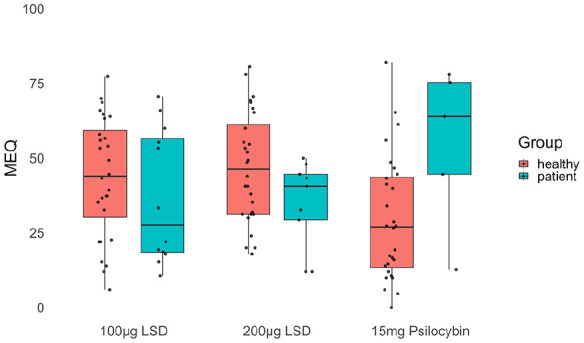
Average MEQ scores at different total doses for healthy subjects and patients. *N* = 28 healthy subjects for all doses and 26 PAT sessions in 19 patients (100 µg LSD = 12, 200 µg LSD = 9, 15 mg psilocybin = 5). There were no statistically significant differences between healthy subjects and patients.

In both samples, most MEQ scores were beneath the criteria for a “complete” mystical experience. Out of 112 dosing sessions in healthy people, only eight resulted in a complete mystical experience, and no PAT sessions resulted in such an experience. Patients and healthy subjects showed no statistical differences in rates of complete mystical experiences (*p* = 0.99) or overall MEQ scores (β = −5.87, SE = 6.40, *p* = 0.58; Figure 5).

**Figure 6.**
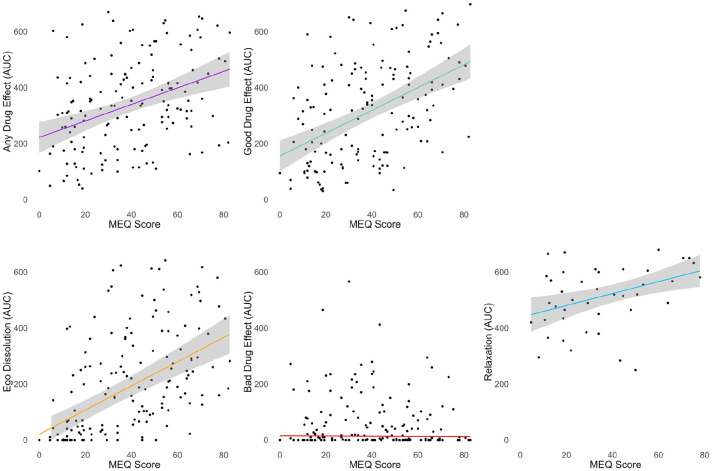
Relationship between real-time ratings of drug effects and subsequent MEQ scores. Gray shading depicts the 95% confidence interval. Ego dissolution was the only significant predictor in a linear mixed model (*p* < 0.001). *N* = 153 dosing sessions in 53 subjects, corresponding statistics from mixed-effects models are found in Table S3. AUC: area under the curve; MEQ; mystical experience questionnaire.

In addition, across both samples, real-time ratings of drug effects explained 55% of the variance in subsequently assessed MEQ scores (*R*^2^_*m*_ = 0.55, *R*^2^_*c*_ = 0.67). Of the real-time ratings, ego dissolution significantly predicted subsequent MEQ scores (β = 0.11, SE = 0.03, *p* < 0.001), but none of the others did (Figure 6, Table S3).

### Antidepressant effects and their relationship to dose, drugs, and subjective effects

**Figure 7.**
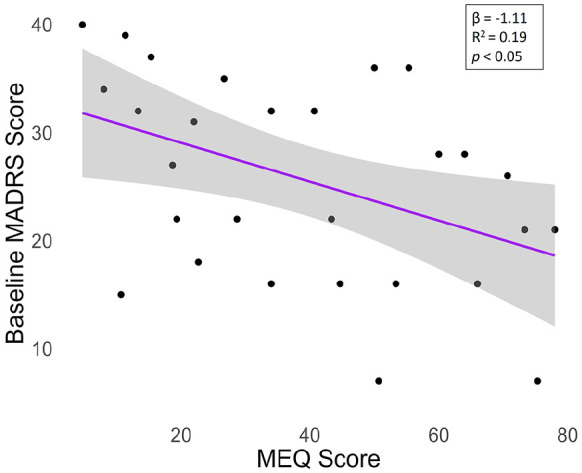
Patients showed significant reductions in MADRS scores after PAT treatment sessions (*p* < 0.001). Data are shown as mean (SEM). *N* = 41 PAT sessions in 21 patients at 8 h and 27 PAT sessions in 14 patients at 7.7 days. MADRS: Montgomery-Åsberg depression rating scale. ; MEQ: mystical experience questionnaire.

In the subset of patients diagnosed with treatment-resistant depression, we investigated changes in MADRS scores after PAT. MADRS scores at baseline and immediately after PAT were available for 41 PAT sessions in 21 of these patients, and MADRS scores at all three timepoints were available for 27 PAT sessions in 14 patients (Table S1). We investigated the effect of PAT on depressive symptoms at both timepoints. On average, patients had MADRS scores of 20.9 points before PAT, corresponding to moderately severe depression. Patients showed a 29% (6.2 points) reduction in MADRS scores immediately after PAT, which was a statistically significant improvement (β = 7.44, SE = 1.42, *p* < 0.001; Figure 7). This improvement was sustained at follow-up, which took place on average 7.7 days after PAT (β = 7.74, SE = 1.64, *p* < 0.001). There was no significant difference in immediate MADRS improvement between patients who did and did not complete a follow-up visit (*p* = 0.65), typically due to logistical reasons (e.g., living far away from the clinic and scheduling issues). Interestingly, improvement in MADRS scores was not dose-dependent immediately after PAT *(*β = −0.01, SE = 0.02, *p* = 0.83*)* or at follow-up (β = 0.04, SE = 0.05, *p* = 0.58). Controlling for dose, there was also no difference in efficacy between LSD and psilocybin immediately after PAT (β = 3.63, SE = 2.59, *p* = 0.43) or at follow-up (β = 0.02, SE = 3.71, *p* = 0.99).

**Figure 8.**
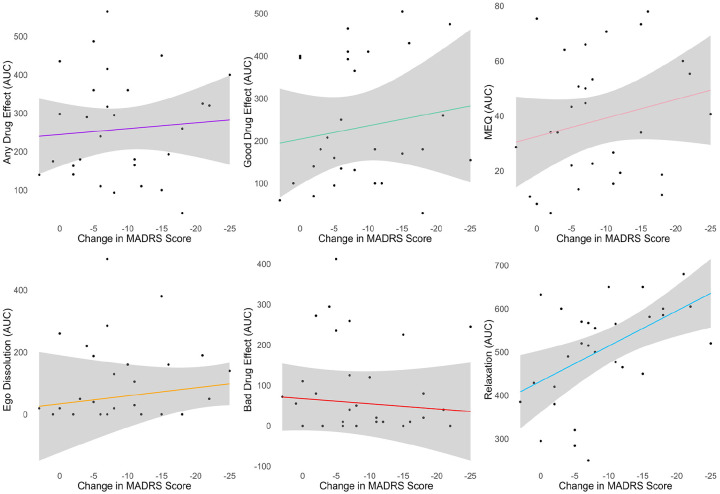
Relationship between acute effects and improvement in depressive symptoms immediately after treatment. Relaxation was the only significant predictor (*p* = 0.03). *N* = 29 treatment sessions in 18 patients, corresponding statistics from the mixed-effects models are shown in Table S5. Gray shading depicts the 95% confidence interval. MADRS: Montgomery–Åsberg depression rating scale; AUC: area under the curve; MEQ: mystical experience questionnaire.

We next investigated the relationship between subjective drug effects and MADRS scores for patients with complete data at each timepoint. Baseline MADRS scores did not significantly predict any of the acute effects (Table S4). Immediately after PAT, we found that all five real-time ratings together explained 28% of the variance in MADRS improvement (*R*^2^_*m*_ = 0.28, *R*^2^_*c*_ = 0.35). Of the real-time ratings, only relaxation predicted MADRS scores in the model, with greater relaxation predicting a greater decrease in MADRS scores (β = −0.04, SE = 0.01, *p* = 0.03; Figure 8, Table S5). By contrast, the MEQ questions predicted 4% of the variance (*R*^2^_*m*_ = 0.04, *R*^2^_*c*_ = 0.04), which was not significant. None of the subjective effects predicted MADRS improvement at the second follow-up.

### Impact of psychiatric medications on subjective psychedelic effects

**Figure 9.**
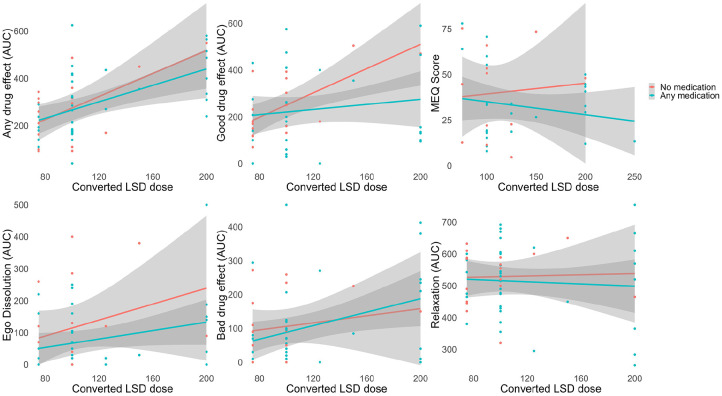
Impact of psychiatric medications on acute drug effects. Controlling for dose, we did not observe significant differences between patients on medication at the time of each PAT session (*N* = 22 patients, 37 sessions) and those not on medication (*N* = 7 patients, 17 sessions). Gray shading represents 95% confidence intervals. AUC: area under the curve; MEQ: mystical experience questionnaire.

Because of the high rate of psychiatric medications in the patient sample, we conducted exploratory analyses to examine the effect of medication on acute drug effects. Controlling for dose, patients who were on medication did not report significantly different acute effects than patients not on psychiatric medication (Figure 9, Table S6). Because of the high rate of polypharmacy, we did not analyze the impact of individual medication types.

### Adverse effects on patients

A summary table of potentially treatment-related side effects in the patient group is shown in Table 2, and side effects for each treatment session are shown in Table S2. Most (93.5%) patients experienced at least one side effect on the scale, the most common being mild headache (32.3% of patients), fatigue (29%), and nausea (29%). The frequency of side effects in the patient sample was similar to those previously reported in healthy subjects using the List of Complaints (Holze et al., 2022b). Headaches and trouble sleeping were treated with over-the-counter medication as needed. Most side effects on the scale were reported to be unpleasant, with a few exceptions, and most patients stated that their side effects did not substantially impact them after the treatment. In all but one case, described below, side effects resolved within 48 h of treatment.

**Table 2.** Frequency and duration of potentially treatment-related adverse effects after psychedelic therapy. Patients were asked whether they had experienced each symptom on the Swiss Psychedelic Side Effects Inventory, as well as how long it lasted, how positive or negative it felt, whether it had a positive or negative impact on their lives, and whether it seemed related to treatment. Ratings of valence and impact were done using a 5-point Likert scale. Data for valence and impact are shown as mean (SD).

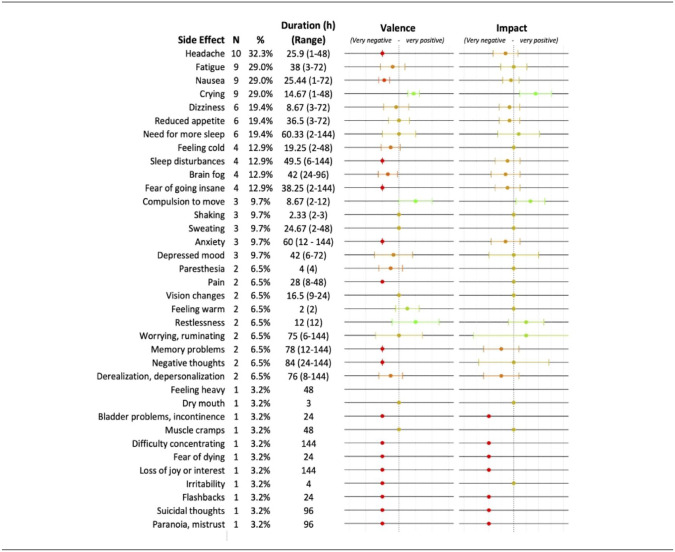

One patient experienced an unusually high number of psychological adverse effects in the first week after his second treatment with 15 mg psilocybin for OCD. The acute experience was challenging, and the patient endorsed relatively high levels of negative drug effects. After the psilocybin effects had dissipated, he reported experiencing anxiety, derealization, non-psychotic hallucinations, and cognitive problems that were likely related to treatment. He also reported passive suicidal thoughts. The patient was treated with intensive outpatient psychotherapy by the treating psychiatrist (GH) with the involvement of the referring physician. All psilocybin-related adverse effects were completely resolved after 6 days, and no changes in medication were necessary. After symptom resolution, the patient continued regular psychotherapy with the treating psychiatrist and without psychedelics, stating that the psychotherapy as a whole had been helpful, though not necessarily because of psilocybin. As of this writing 18 months post-PAT, no long-term adverse effects from the psilocybin episode have manifested.

## Discussion

This study presents insights from the real-world clinical practice of PAT as part of the Swiss limited medical use program and compares the subjective effects of LSD and psilocybin in patients and healthy subjects. Compared to healthy subjects, patients showed similar scores on measures of overall drug effects and mystical-type experiences. However, patients also experienced less ego dissolution, as well as an increase in bad drug effects which was not statistically significant but may be worth investigating in future studies.

Like this study, a previous comparison of LSD effects in patients and healthy subjects found no differences in MEQ scores (Schmid et al., 2021). The study also investigated differences in scores on the 5D-ASC (Dittrich, 1998), showing that patients tended to score lower on a few subscales (changed meaning of percepts, visionary restructuralization, and audio-visual synesthesia), despite an overall similarity in LSD effects (Schmid et al., 2021). One possible explanation for the differences in acute effects between patients and healthy subjects in our sample is the presence of psychiatric medications, which were primarily SSRIs and other antidepressants. Though most patients do not take their usual medications on the morning of PAT sessions, antidepressants cause long-term adaptations in the brain that can take weeks or months to normalize after discontinuation (Horowitz et al., 2023). We found no effect of psychiatric medications within the patient sample, but it is possible that our analysis was underpowered. Aside from medications, it is also possible that the patients in psychotherapy were confronted with more anxiety and difficult psychological material than healthy people in a basic research setting, resulting in higher ratings of bad drug effects and perhaps reduced willingness to give up ego-related control and allow ego boundaries to dissolve. This highlights the frequent clinical observation that patients may need particularly intensive supportive care during, as well as after psychedelic experiences (Carhart-Harris et al., 2018). In addition, people with higher scores on the personality trait neuroticism may be prone to more severe challenging experiences from psychedelics (i.e., bad drug effects), and neuroticism is closely related to symptoms of anxiety and depression (Barrett et al., 2017; Ormel et al., 2013).

Interestingly, we also found a positive relationship between age and the experience of ego dissolution. To our knowledge, this is the first report of a relationship between age and psychedelic-induced ego dissolution, but previous research suggested a general trend toward reduced psychedelic drug effects (Aday et al., 2021) or fewer impairments of control and cognition with advancing age (Studerus et al., 2012). Notably, psilocybin and LSD induce subjective effects via the serotonin 2A receptor (Kometer et al., 2013; Preller et al., 2017), the expression of which declines steadily with age (Versijpt et al., 2003). Lower expression of serotonin 2A receptors was found to be associated with greater peak duration of acute drug effects and higher MEQ scores after psilocybin treatment, though ego dissolution was not measured specifically (Stenbaek et al., 2021). Future studies in larger samples with a broad age range could elucidate whether subjective psychedelic effects meaningfully change with age.

Though results from open-label treatments should be interpreted with caution, our data support the idea that PAT with LSD and psilocybin has a rapid antidepressant effect and is safe in a real-world clinical setting. Importantly, psilocybin and LSD were similarly effective in improving depressive symptoms, consistent with their similar acute effects in healthy subjects and controlled clinical studies in patients (Holze et al., 2022b, 2024). In addition, no antidepressant-related safety concerns arose in patients who skipped a single dose of antidepressant medication on the morning of PAT without otherwise tapering off. This is encouraging because tapering off of antidepressants can cause unpleasant withdrawal symptoms and increase the risk of future depressive episodes, which presents a barrier to the use of PAT for many patients. Consistently, escitalopram and psilocybin co-administration were well tolerated (Becker et al., 2022). A previous study of 19 patients who did not wish to taper off of their SSRIs before psilocybin-assisted therapy also reported no negative effects on safety, though it was an uncontrolled study (Goodwin et al., 2023). It may be some patients undergoing PAT may not need to stop taking their antidepressants, but larger samples and controlled studies are needed to confirm this hypothesis.

We did not find an impact of psychiatric medications, which were primarily antidepressants, on acute drug effects. Though the high rate of polypharmacy severely limited our analysis, most patients were taking medications that are thought to reduce psychedelic drug effects. There is some evidence that SSRIs blunt unpleasant drug effects from psychedelics, though they do not appear to reduce the overall effect (Becker et al., 2022). In addition, both antipsychotics and benzodiazepines can reduce the acute effects of psychedelics (Johnson et al., 2008). Previous research has involved participants who took psychedelics concomitantly with other psychiatric medications, and in our sample, most participants had skipped their scheduled dose on the morning of PAT. It is possible that the residual effects of SSRIs, antipsychotics, and benzodiazepines may be minimal if patients undergoing PAT skip a dose before their PAT session. However, it is also possible that the high rate of medication use in our patient sample explains why some psychedelic drug effects were reduced compared to healthy subjects. Controlled studies are needed to determine the impact of different psychiatric medications on psychedelic drug effects relevant to PAT.

The data on adverse effects highlight the essential role of psychological support in PAT, particularly for patients with severe, treatment-refractory conditions. Overall, adverse effects were short-lived, mild, and comparable to those seen in healthy subjects (Holze et al., 2022a, 2022b). However, one patient experienced severe, psilocybin-related symptoms which lasted for several days, including anxiety, derealization, non-psychotic hallucinations, and passive suicidal ideation. Controlled clinical trials with psilocybin, as well as other psychedelics, have occasionally reported increases in suicidality after treatment, sometimes with extremely brief information about treatment-relatedness (Breeksema et al., 2022; Goodwin et al., 2022). Furthermore, similar post-trip difficulties have been reported in the literature outside of clinical settings (Evans et al., 2023; Simonsson et al., 2023), and according to one report, 7.6% of people who had a challenging psilocybin experience reported psychological difficulties requiring professional support (Carbonaro et al., 2016). In the current case, we wish to emphasize the vital role of intensive therapeutic support and a strong therapeutic alliance for dealing with post-trip difficulties (Levin et al., 2024). Without proper support, we find it unlikely that the patient would have recovered as quickly and completely as he did. It is important to acknowledge that though they may be relatively rare in controlled settings, post-trip difficulties can still occur. It is essential for patients’ well-being that proper support is readily available, particularly given that the few days after PAT may constitute a particularly sensitive “window of neuroplasticity” (Calder and Hasler, 2023b).

Interestingly, we saw no signs of a dose–response relationship for antidepressant effect in the dose range analyzed. Given the dose–response effects reported by recent meta-analyses with greater dose ranges and sample sizes, we would not draw strong conclusions from this (Perez et al., 2023). However, other factors than the dose may also contribute to the success of PAT. The therapeutic value of relatively low doses of psilocybin and LSD (e.g., <30 mg or <150 µg, respectively) has long been noted among practitioners of psycholytic therapy. While psychedelic therapy seeks to induce ecstatic mystical-type experiences, including the dissolution of ego boundaries, psycholytic therapy seeks to activate therapeutically useful psychodynamic processes while leaving some ego structures intact (Passie et al., 2022). The relatively low levels of mystical experience and ego dissolution in this patient sample are more reminiscent of psycholytic than psychedelic effects.

Mystical-type experiences did not predict antidepressant effects, and for both healthy people and patients, “complete” mystical experiences were rare even at doses considered quite high, that is, 200 µg LSD base and 30 mg psilocybin. Volunteers in other studies using psilocybin may have been more spiritually inclined, perhaps leading to greater effects on mystical-type experiences (Liechti et al., 2017). In the patient group, these findings are consistent with previous research showing that “complete” mystical experiences are rare and not necessary for therapeutic effect (Gasser et al., 2015; Schmid et al., 2021), although other studies do show correlations between MEQ30 scores and therapeutic effects in PAT for depression and anxiety disorders (Davis et al., 2021; Griffiths et al., 2016; Holze et al., 2023b; Ross et al., 2016; Roseman et al., 2017). We find it plausible that other psychedelic effects may also underlie therapeutic responses. In this sample, the limited role of mystical experience may be explained by the focus on biographical, trauma-related, and challenging content in the treatment of patients with severe forms of depression (Hasler, 2022), as well as the fact that patients may have sought treatment without a specific interest in spiritual healing.

The real-time ratings of acute drug effects collectively explained 29% of the variance in MADRS improvement immediately after PAT, while the MEQ explained only 4%. Furthermore, real-time effect ratings explained a large proportion (55%) of the variance in subsequent MEQ scores. Taken together, these data suggest that real-time drug effect ratings may be more accurate and parsimonious predictors of immediate clinical improvement than retrospective MEQ scores. Monitoring drug effects in real time, in particular relaxation, may thus help optimize the therapeutic process during PAT sessions.

The potential role of relaxation during PAT deserves further discussion, as it emerged as a particularly strong predictor of antidepressant response. The importance of mental and physical relaxation in PAT has been noted in qualitative accounts from patients before (Gasser et al., 2015). Outside of PAT several types of relaxation therapy, including progressive muscle relaxation and meditation techniques, have been shown to help treat depression and may be similarly effective to psychotherapy (Jia et al., 2020; Jorm et al., 2008). The ability to relax oneself, especially in stressful situations, may be an important coping mechanism that promotes feelings of self-efficacy, which are often reduced in depression (Fung and White, 2012). In addition, a study of people combining psychedelics with meditation identified feelings of deep relaxation and peacefulness as helpful for navigating psychedelic experiences, as well as meaningful aspects of the experience itself (Azmoodeh et al., 2023). Relaxation may also reflect feelings of comfort and safety with the setting, which has been associated with more positive effects on well-being in previous studies (Golden et al., 2022; Haijen et al., 2018). It is also possible that greater relaxation goes together with “letting go” and allowing the psychedelic experience to take its course, creating more favorable conditions for facing difficult psychological material, important learning experiences, trauma processing, and emotional breakthroughs (Wolff et al., 2020; Roseman et al., 2019; Zeifman et al., 2023). Excessive tension or anxiety during a psychedelic experience, by contrast, can inhibit these processes. As noted in some manuals for conducting psychedelic sessions (Haden, 2019), it may be helpful for patients undergoing psychedelic therapy to begin their sessions with relaxation techniques, such as breathing exercises, progressive muscle relaxation, or meditation, which allow them to take full advantage of the psychedelic drug effects. Ideally, these are taught in preparatory sessions before PAT so that patients are familiar with them before entering an altered state.

### Limitations

This data should be interpreted in the context of its limitations. One major limitation of this study is the heterogeneity of naturalistic data. Patients undergoing PAT had different clinical characteristics, medications, doses, and treatment durations. While this may increase generalizability to real-world clinical practice, it also means conclusions should be drawn with caution. In addition, the patient data came from an open-label, non-randomized, uncontrolled study and all patients were in psychotherapy before and after dosing sessions, thus making it difficult to establish the causal effects of PAT. Furthermore, the follow-up times were relatively short, and this data cannot be used to conclude long-term efficacy. In addition, the relatively small patient sample was underpowered for some analyses, and some data were missing for follow-up MADRS scores and the MEQ. For some doses of LSD psilocybin (e.g., 150 µg LSD, 25 mg psilocybin), the sample size was extremely small and thus did not allow for clear inferences about dose–response effects. In addition, the flexible dosing regimen may limit the comparability of different doses, because only patients who did not respond adequately to a lower dose received a larger one. The fact that the healthy and patient samples came from two different hospitals may have also introduced some variation in subjective psychedelic effects. Psychedelic experiences vary depending not only on individual characteristics, such as health status, but also on the type of preparation available, intentions agreed upon before the experience, and the environment in which the experience takes place (Carhart-Harris et al., 2018; Golden et al., 2022).

## Conclusions

Taken together, these findings suggest that PAT with LSD and psilocybin is safe for a heterogeneous group of patients in a real-world clinical setting which includes supportive psychotherapy, and that patients may experience a slightly different profile of subjective drug effects than healthy controls. There may be no need for most patients with treatment-resistant depression, including those with comorbidities, to discontinue medications before undergoing PAT. In addition, simple ratings of drug effects during PAT sessions may be better predictors of immediate clinical outcomes than retrospective MEQ scores, and further research is warranted regarding the potentially important role of relaxation.

## Supplemental Material

sj-docx-1-jop-10.1177_02698811241278873 – Supplemental material for Naturalistic psychedelic therapy: The role of relaxation and subjective drug effects in antidepressant response Supplemental material, sj-docx-1-jop-10.1177_02698811241278873 for Naturalistic psychedelic therapy: The role of relaxation and subjective drug effects in antidepressant response by Abigail E Calder, Benjamin Rausch, Matthias E Liechti, Friederike Holze and Gregor Hasler in Journal of Psychopharmacology

sj-docx-2-jop-10.1177_02698811241278873 – Supplemental material for Naturalistic psychedelic therapy: The role of relaxation and subjective drug effects in antidepressant response Supplemental material, sj-docx-2-jop-10.1177_02698811241278873 for Naturalistic psychedelic therapy: The role of relaxation and subjective drug effects in antidepressant response by Abigail E Calder, Benjamin Rausch, Matthias E Liechti, Friederike Holze and Gregor Hasler in Journal of Psychopharmacology

sj-jpg-3-jop-10.1177_02698811241278873 – Supplemental material for Naturalistic psychedelic therapy: The role of relaxation and subjective drug effects in antidepressant response Supplemental material, sj-jpg-3-jop-10.1177_02698811241278873 for Naturalistic psychedelic therapy: The role of relaxation and subjective drug effects in antidepressant response by Abigail E Calder, Benjamin Rausch, Matthias E Liechti, Friederike Holze and Gregor Hasler in Journal of Psychopharmacology

sj-jpg-4-jop-10.1177_02698811241278873 – Supplemental material for Naturalistic psychedelic therapy: The role of relaxation and subjective drug effects in antidepressant response Supplemental material, sj-jpg-4-jop-10.1177_02698811241278873 for Naturalistic psychedelic therapy: The role of relaxation and subjective drug effects in antidepressant response by Abigail E Calder, Benjamin Rausch, Matthias E Liechti, Friederike Holze and Gregor Hasler in Journal of Psychopharmacology
