# Supplementary material for: Naturalistic psychedelic therapy: The role of relaxation and subjective drug effects in antidepressant response
Source: J Psychopharmacol. 2024 Sep 20;38(10):873–86. doi: 10.1177/02698811241278873 (PMC11487903; doi:10.1177/02698811241278873)
Supplement: sj-docx-1-jop-10.1177_02698811241278873 – Supplemental material for Naturalistic psychedelic therapy: The role of relaxation and subjective drug effects in antidepressant response [file sj-docx-1-jop-10.1177_02698811241278873.docx]

# Supplementary Methods

Supplement to: *Naturalistic psychedelic therapy: The role of relaxation and subjective drug effects in antidepressant response.* Calder AE, Rausch B, Liechti ME, Holze F & Hasler G.

## Handling of second doses

In the patient sample, some patients received either a second dose of LSD or psilocybin (N = 16 sessions) or a sedative (N = 3 sessions) between one and three hours after the initial dose. To prevent this from biasing our analyses of acute drug effects, we did not exclude these patients but treated all timepoints after the second dose as missing data for some analyses. Missing data required for calculating the AUC was imputed using mean imputation by group, drug, and dose.

Based on the assumptions of each analysis, we either used 1) the data in its original form, including data from after second doses, or 2) data which imputed all timepoints after second doses. This is described in more detail for each analysis below.

## Real-time ratings of subjective effects in patients and healthy subjects

When assessing the dose-response relationships for real-time ratings in both patients and healthy subjects, we used the imputed data. This was because we would not expect dose-response curves to be the same if a dose is given all at once or in two halves 1-3 hours apart.

## Mystical experiences in patients and healthy subjects

When analyzing MEQ data in patients and healthy subjects, we used the original data. The MEQ asks subjects to retrospectively rate the maximum intensity of 30 items at any point during a dosing session. Thus, the total dose received is likely more relevant than the initial dose, particularly because most second doses were given within the window of peak effects.

## Antidepressant effects and their relationship to dose, drugs, and subjective effects

When analyzing whether baseline MADRS scores could predict real-time ratings of acute drug effects, we used the imputed data. This was done to avoid the confounding effect of second doses as additional predictors of response.

When analyzing whether baseline MADRS scores could predict MEQ scores, we used the original data for the reasons stated above.

When analyzing whether acute drug effects predicted post-treatment MADRS and MEQ scores, we used the original data. This was because we were interested in the impact of the actual subjective effects experienced, regardless of the dose given.

## Impact of psychiatric medications on subjective psychedelic effects

When analyzing the impact of SSRIs and other medications, we used the imputed data to avoid the confounding effect of second doses as additional predictors of subjective effects. For the MEQ, we used the original data for the reasons stated above.
