# Supplementary material for: Naturalistic psychedelic therapy: The role of relaxation and subjective drug effects in antidepressant response
Source: J Psychopharmacol. 2024 Sep 20;38(10):873–86. doi: 10.1177/02698811241278873 (PMC11487903; doi:10.1177/02698811241278873)
Supplement: sj-docx-2-jop-10.1177_02698811241278873 – Supplemental material for Naturalistic psychedelic therapy: The role of relaxation and subjective drug effects in antidepressant response [file sj-docx-2-jop-10.1177_02698811241278873.docx]

# Supplementary Tables S3-S6

Supplement to: *Naturalistic psychedelic therapy: The role of relaxation and subjective drug effects in antidepressant response.* Calder AE, Rausch B, Liechti ME, Holze F & Hasler G.

*Table S3. Results of linear mixed effects models analyzing the relationship between real-time ratings and subsequent MEQ scores. N = 153 dosing sessions in 53 subjects. *** corrected p <0.001.*

|  | **Estimate (β)** | **Std. Error** | **df** | **t** | ***p*** |
| --- | --- | --- | --- | --- | --- |
| Any effect | -0.06 | 0.03 | 28.12 | -1.97 | 0.20 |
| Good effect | 0.06 | 0.02 | 24.56 | 2.56 | 0.09 |
| Bad effect | 0.03 | 0.02 | 34.88 | 1.21 | 0.55 |
| Ego dissolution | 0.11 | 0.02 | 34.25 | 4.68 | **0.0005***** |
| Relaxation | 0.03 | 0.02 | 31.14 | 1.15 | 0.55 |

*Table S4. Results of linear mixed effects models analyzing the relationship between depression severity at baseline and subsequent acute drug effects. No significant effects were found. N = 41 PAT sessions in 21 patients.*

|  | **Estimate (β)** | **Std. Error** | **df** | **t** | ***p*** |
| --- | --- | --- | --- | --- | --- |
| Any effect | -2.13 | 2.18 | 38.45 | -0.98 | 0.58 |
| Good effect | -1.48 | 2.27 | 36.74 | -0.65 | 0.65 |
| Bad effect | -1.63 | 1.86 | 37.18 | -0.87 | 0.58 |
| Ego dissolution | -1.98 | 2.04 | 39.00 | -0.97 | 0.58 |
| Relaxation | -2.90 | 1.79 | 38.94 | -1.62 | 0.33 |
| MEQ | -0.78 | 0.40 | 24.65 | -1.94 | 0.21 |

*Table S5. Results of linear mixed effects models testing the relationship between acute drug effects and reduction in depressive symptoms. Negative estimates indicate a positive effect on symptom reduction. Relaxation during treatment sessions significantly predicted initial antidepressant response, but no other significant effects were found. For each timepoint, only complete cases were used. MADRS = Montgomery–Åsberg Depression Rating Scale, MEQ = Mystical Experience Questionnaire. *p < 0.05.*

|  |  | **Estimate (β)** | **Std. Error** | **df** | ***t*** | ***p*** |
| --- | --- | --- | --- | --- | --- | --- |
| Post-PAT (N =  29 sessions, 18 patients) | Any effect | -0.03 | 0.02 | 20.08 | -1.54 | 0.38 |
|  | Good effect | 0.02 | 0.02 | 16.32 | 1.21 | 0.54 |
|  | Bad effect | 0.02 | 0.02 | 21.89 | 1.00 | 0.58 |
|  | Ego dissolution | 0.00 | 0.01 | 18.27 | -0.12 | 0.95 |
|  | Relaxation | -0.04 | 0.01 | 22.01 | -3.12 | **0.03*** |
|  | MEQ | -0.07 | 0.06 | -7.00 | 1.10 | 0.58 |
| Follow up 7.7 days (N =  19 sessions 11 patients) | Any effect | 0.03059 | 0.03969 | 10 | 0.771 | 0.64 |
|  | Good effect | -0.02224 | 0.03145 | 10 | -0.707 | 0.65 |
|  | Bad effect | 0.02694 | 0.03741 | 10 | 0.72 | 0.65 |
|  | Ego dissolution | -0.02338 | 0.05008 | 10 | -0.467 | 0.74 |
|  | Relaxation | 0.04312 | 0.03879 | 10 | 1.112 | 0.57 |
|  | MEQ | -0.05 | 0.09 | 14.00 | -0.53 | 0.72 |

*Table S6. Impact of any psychiatric medication on acute drug effects. Twenty-two patients (37 sessions) were taking psychiatric medication before undergoing PAT, and 7 patients (17 sessions) were not on psychiatric medication. There was no significant effect.*

|  | **Sum Sq** | **Mean Sq** | **NumDf** | **DenDf** | **F** | ***p*** |
| --- | --- | --- | --- | --- | --- | --- |
| Any effect | 4349.00 | 4349.00 | 1.00 | 31.68 | 0.72 | 0.58 |
| Good effect | 218.00 | 218.00 | 1.00 | 26.68 | 0.01 | 0.95 |
| Bad effect | 4304.00 | 4304.00 | 1.00 | 17.08 | 0.41 | 0.65 |
| Ego dissolution | 7979.00 | 7979.00 | 1.00 | 27.92 | 0.92 | 0.58 |
| Relaxation | 747.19 | 747.19 | 1.00 | 25.27 | 0.10 | 0.83 |
| MEQ | 141.66 | 141.66 | 1.00 | 28.39 | 0.72 | 0.58 |
