# Supplementary figures and images for: Naturalistic psychedelic therapy: The role of relaxation and subjective drug effects in antidepressant response
Source: J Psychopharmacol. 2024 Sep 20;38(10):873–86. doi: 10.1177/02698811241278873 (PMC11487903; doi:10.1177/02698811241278873)

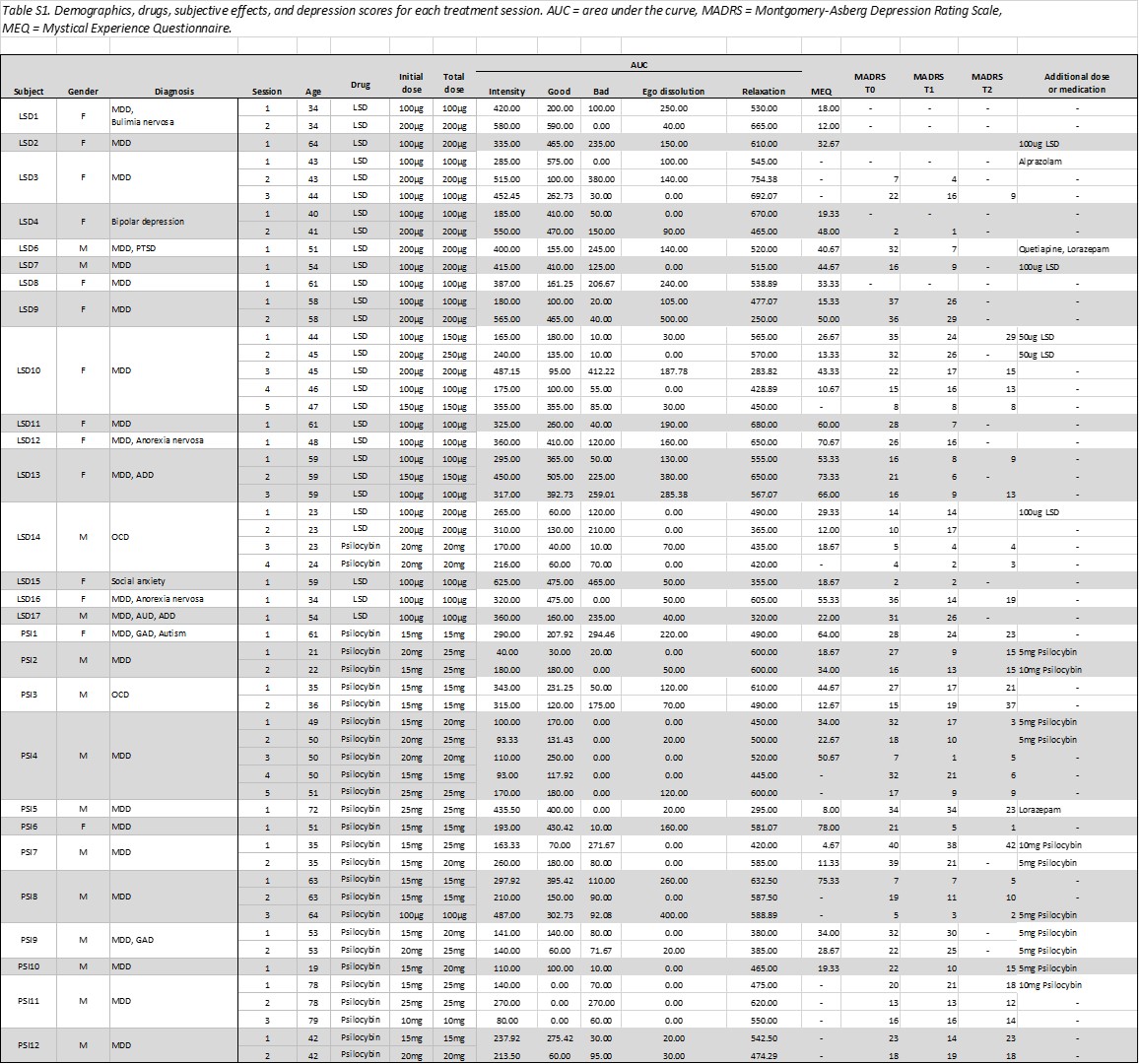

Supplement: sj-jpg-3-jop-10.1177_02698811241278873 – Supplemental material for Naturalistic psychedelic therapy: The role of relaxation and subjective drug effects in antidepressant response [file sj-jpg-3-jop-10.1177_02698811241278873.jpg]

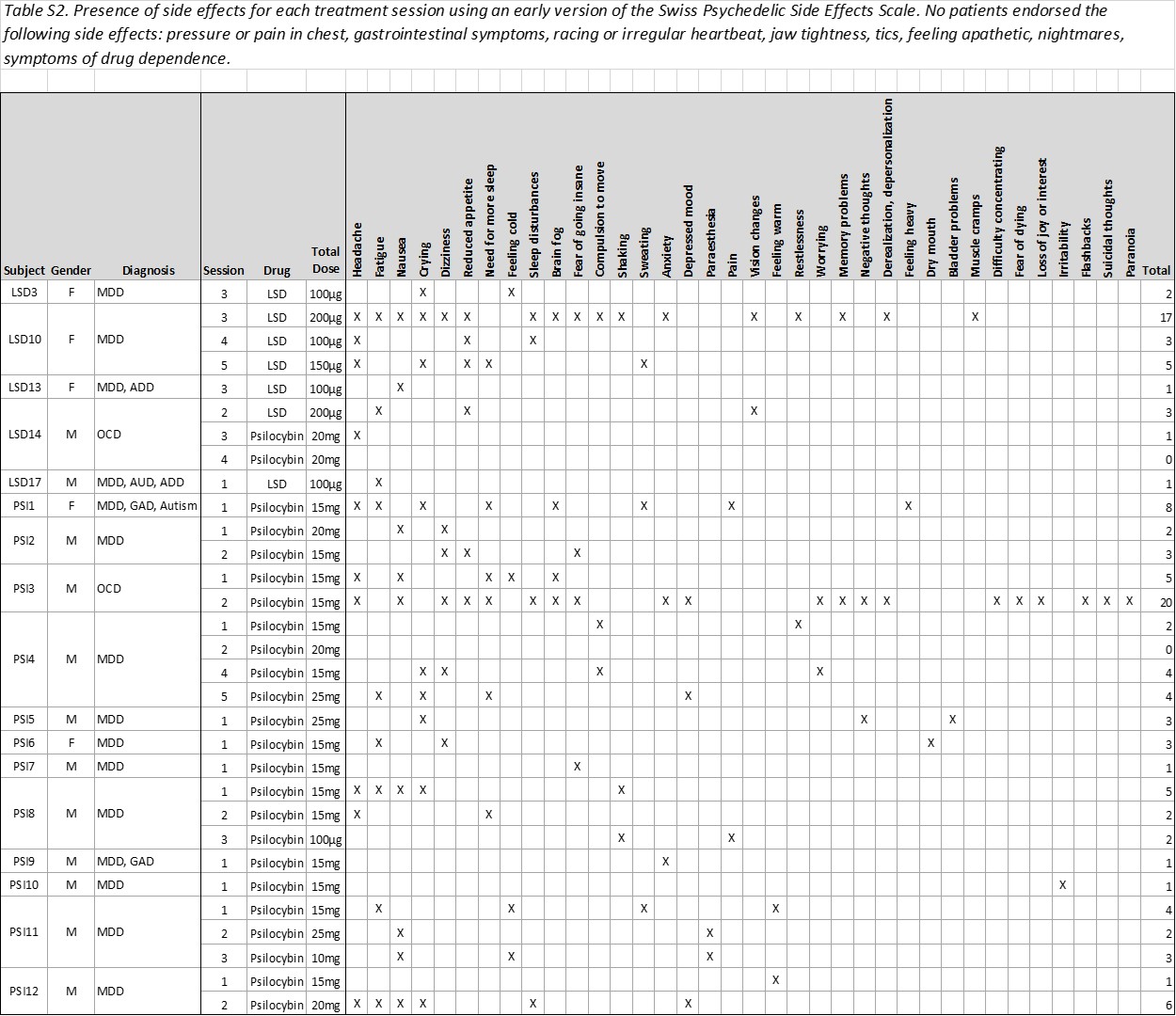

Supplement: sj-jpg-4-jop-10.1177_02698811241278873 – Supplemental material for Naturalistic psychedelic therapy: The role of relaxation and subjective drug effects in antidepressant response [file sj-jpg-4-jop-10.1177_02698811241278873.jpg]
